# Supplementary material for: The micro-economic effects of COVID-19 containment measures: A simple model and evidence from China
Source: PLoS One. 2023 Jul 17;18(7):e0288632. doi: 10.1371/journal.pone.0288632 (PMC10351727; doi:10.1371/journal.pone.0288632)
Supplement: S1 Table — (PDF) [file pone.0288632.s001.pdf]

S1 Table. Descriptive statistics of COVID-19 data in prefecture-level cities in China (excluding imported or unconfirmed cases)

Month	Variable	Mean	Standard deviation	Min	Median	Max	Month	Variable	Mean	Standard deviation	Min	Median	Max
January	Cumulative cases	21.205	121.740	0	4	3215	April	Cumulative cases	221.742	2601.636	0	15	50,333
	Cumulative recoveries	0.359	3.791	0	0	103		Cumulative recoveries	207.413	2421.417	0	14	47,283
	Cumulative deaths	0.484	6.799	0	0	192		Cumulative deaths	10.545	167.610	0	0	3869
	New cases	5.564	30.188	-55	1	892		New cases	0.100	3.281	-2	0	326
	New recoveries	0.078	0.913	-4	0	28		New recoveries	0.192	9.818	-948	0	183
	New deaths	0.126	1.478	-1	0	33		New deaths	0.118	12.172	0	0	1290
	Mortality	0.449	0.468	0	0.143	1		Mortality	0.014	0.040	0	0	0.333
February	Cumulative cases	167.531	1935.698	0	15	48,557	May	Cumulative cases	222.709	2609.015	0	15	50,340
	Cumulative recoveries	38.057	384.628	0	4	17,656		Cumulative recoveries	209.818	2409.446	0	14	46,467
	Cumulative deaths	4.708	74.721	0	0	2169		Cumulative deaths	12.383	199.741	0	0	3869
	New cases	6.943	154.668	-107	0	13,436		New cases	0.013	0.250	-1	0	13
	New recoveries	4.019	47.480	-19	0	2379		New recoveries	0.047	0.606	0	0	21
	New deaths	0.264	4.380	-1	0	216		New deaths	0.000	0.013	0	0	1
	Mortality	0.037	0.136	0	0	1		Mortality	0.013	0.040	0	0	0.333
March	Cumulative cases	217.248	2590.624	0	15	50,006	January-May	Cumulative cases	199.496	2406.656	0	14	50,340
	Cumulative recoveries	173.312	1933.651	0	14	46,002		Cumulative recoveries	153.406	1955.735	0	10	47,283
	Cumulative deaths	8.506	126.400	0	0	2548		Cumulative deaths	8.763	147.912	0	0	3869
	New cases	0.254	6.306	-12	0	565		New cases	1.794	71.451	-107	0	13,436
	New recoveries	3.182	53.547	-12	0	1878		New recoveries	1.708	34.951	-948	0	2379
	New deaths	0.042	0.792	-1	0	32		New deaths	0.100	6.354	-1	0	1290
	Mortality	0.014	0.040	0	0	0.333		Mortality	0.020	0.084	0	0	1

Note: Negative values of the number of new cases may be due to changes in quarantine results (e.g., retesting positive during quarantine) or inter-regional transfer of patients. In particular, only 31/22,814 of the samples had a negative number of new cases in March-April. The number of new recoveries of -948 was due to data correction for Wuhan on April 17 .
