# Supplementary material for: The micro-economic effects of COVID-19 containment measures: A simple model and evidence from China
Source: PLoS One. 2023 Jul 17;18(7):e0288632. doi: 10.1371/journal.pone.0288632 (PMC10351727; doi:10.1371/journal.pone.0288632)
Supplement: Attachment — Submitted filename: D23-06635 Report_SA.pdf [file pone.0288632.s002.pdf]

PONE-D-23-06635

The Micro-Economic Effects of COVID-19 Containment Measures:
A Simple Model and Evidence from China

Referee Report

General Remarks

This paper wishes to disentangle the negative economic impact of the pandemic lockdown policy, dubbing it 'the shutdown effect' (SDE) from the presumptive benefits, the 'anti-epidemic effect' (AEE) in the context of the COVID containment measures in China. The authors make use of the 'Susceptible-Infected-Recovered/Removed' (SIR) model from the epidemiology literature to examine the trade-off between the two consequences of the lockdown policy in the anticipation that one can determine when it may be optimal to lift the lockdown policy. Ideally this would require the determination of the critical point where the social value of the shutdown losses matches the social value of AEE.

The SIR model is an analytic device that is typically employed to solve an optimal control problem, which has been analysed by many, but the present authors rely on a recent paper by Alvarez et al (2021). However, instead of cross-country analysis, they model the COVID situation in the context of China with micro data (enterprise level). Morbidity data came a panel of prefecture level cities.

The analysis seems comprehensive, and the authors generate valuable policy suggestions on the timing of the enforcement and withdrawal of the lock down policy, on the scope of testing and related preventive/mitigating public health measures as well as social interventions in ensuring the sustenance of daily existence. They also explore the scope of digital interventions where physical contacts and interventions are not practicable.

Recommendation

I recommend publication of the paper after suitable revisions as outlined below under the Specific Comments. These are mainly in the nature of conceptual issues that need to be clarified without adding to the length of the final paper from the current length.

Specific Comments

(1) *Value of Statistical life*: While in the major western countries, there exist a good body of analytical work estimating the value of a statistical life, a key ingredient in valuing the social value of anti-epidemic effects. I am not aware of any studies of this nature in the Chinese context. How did the authors get around this?

(2) *Population Heterogeneity*: Naturally in any context, the universe of the underlying population at risk, i.e., the susceptible, would exhibit heterogeneity in the shape of

natural immunity from lifestyle/occupational hazards, exposure to prior health/viruses, and of course there are those who are immune compromised in various degrees. How does a panel data of morbidity allow analysts to model the heterogeneity in empirical work? What are the pitfalls in ignoring this element?

(3) *Quality of data*: The analysis undertaken here is data intensive, which implies that a similar analysis cannot be done in every context, e.g., in the context of countries with limited resources and skill shortages. In western media, perhaps unfairly, there has been doubts on the quality of Chinese epidemiological data, especially during the early COVID onslaught in Wuhan. Some mention ought to be made of what was done to cross-check /validate the data used in the analysis performed here.

(4) *Testing & Quarantine*: The authors report that "the proportion of licensed physicians to the urban population is used to measure the quantity of testing and quarantine resources, the average size of hospitals (number of doctors/number of hospitals) to measure the quality of testing and quarantine resources …". A recognition ought to be made that these means may not be suitable in contexts due to the physical constraints (e.g., due to supply chain issues) on the supply of kits/essential devices had been indifferent in different countries, and often within a country between central cities and the regional centres.

Composition, Grammar and Style Issues:

(i) References: The authors should follow a standard reference format, e.g., U Chicago guidelines applicable to published and unpublished/web material.

(ii) Grammar: This is a well written paper.
